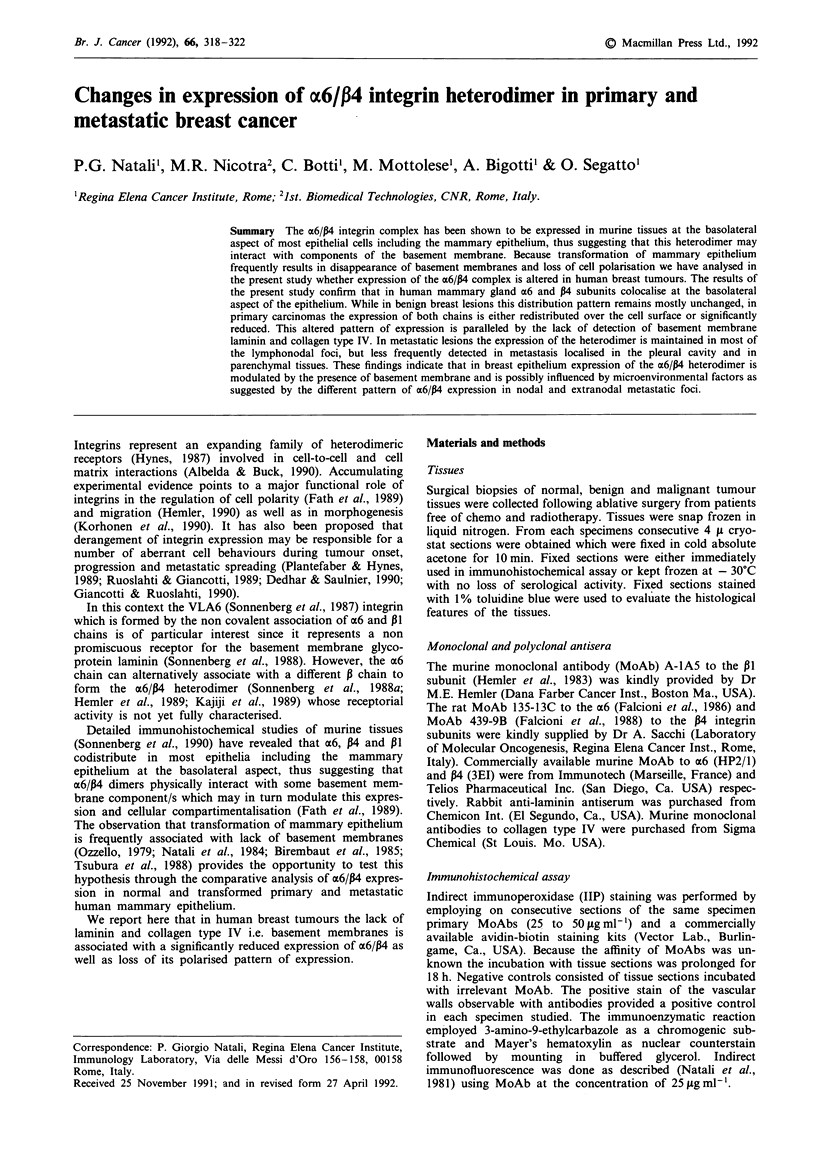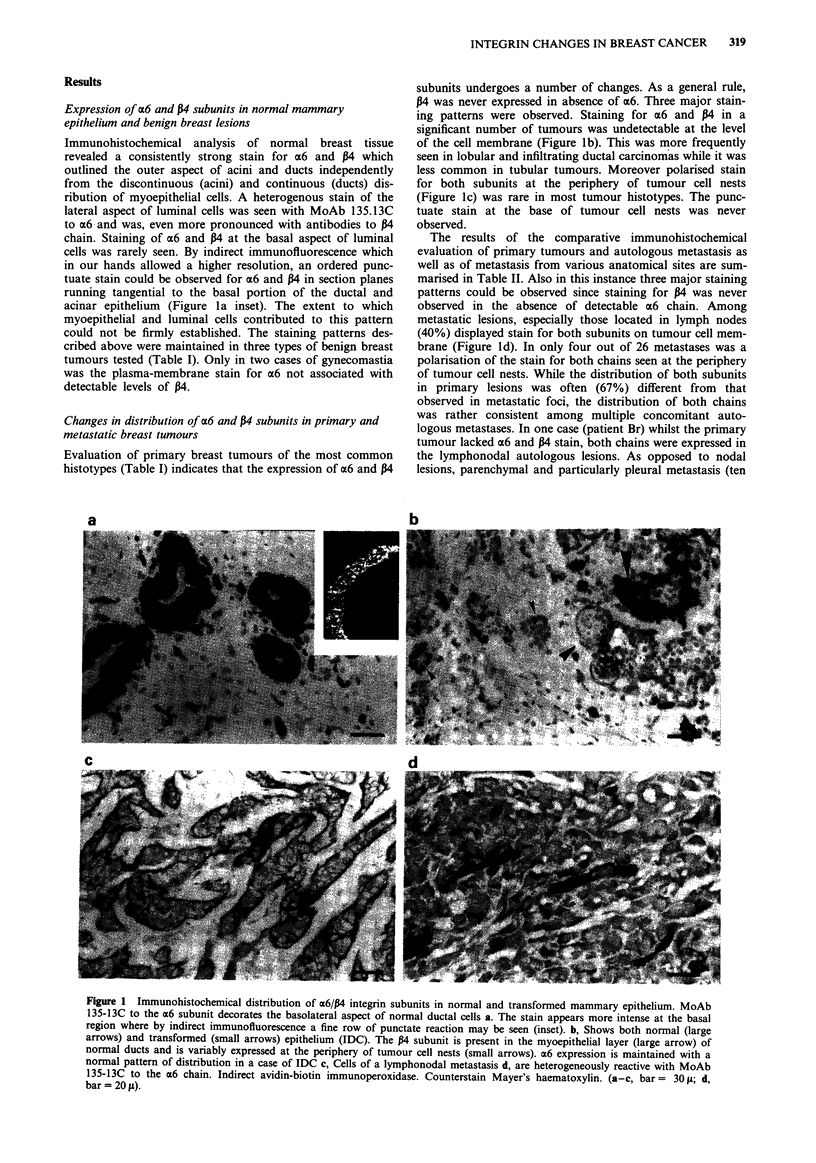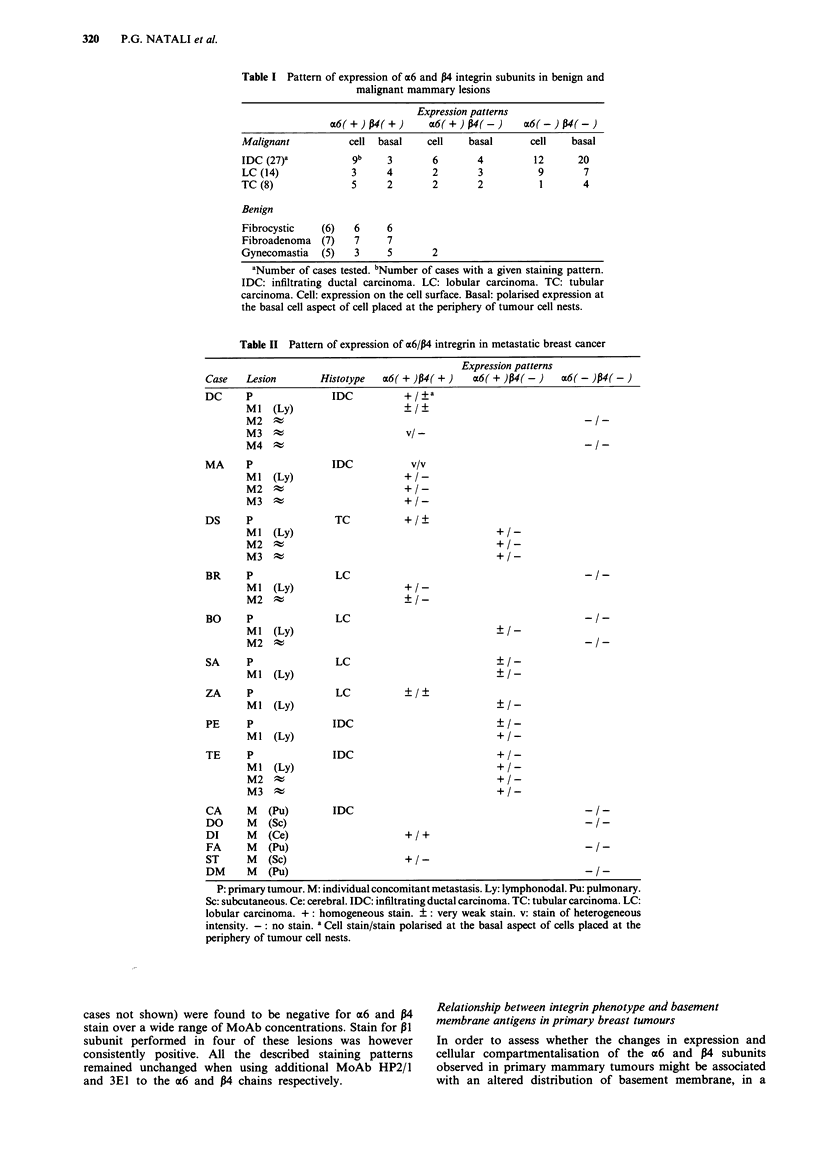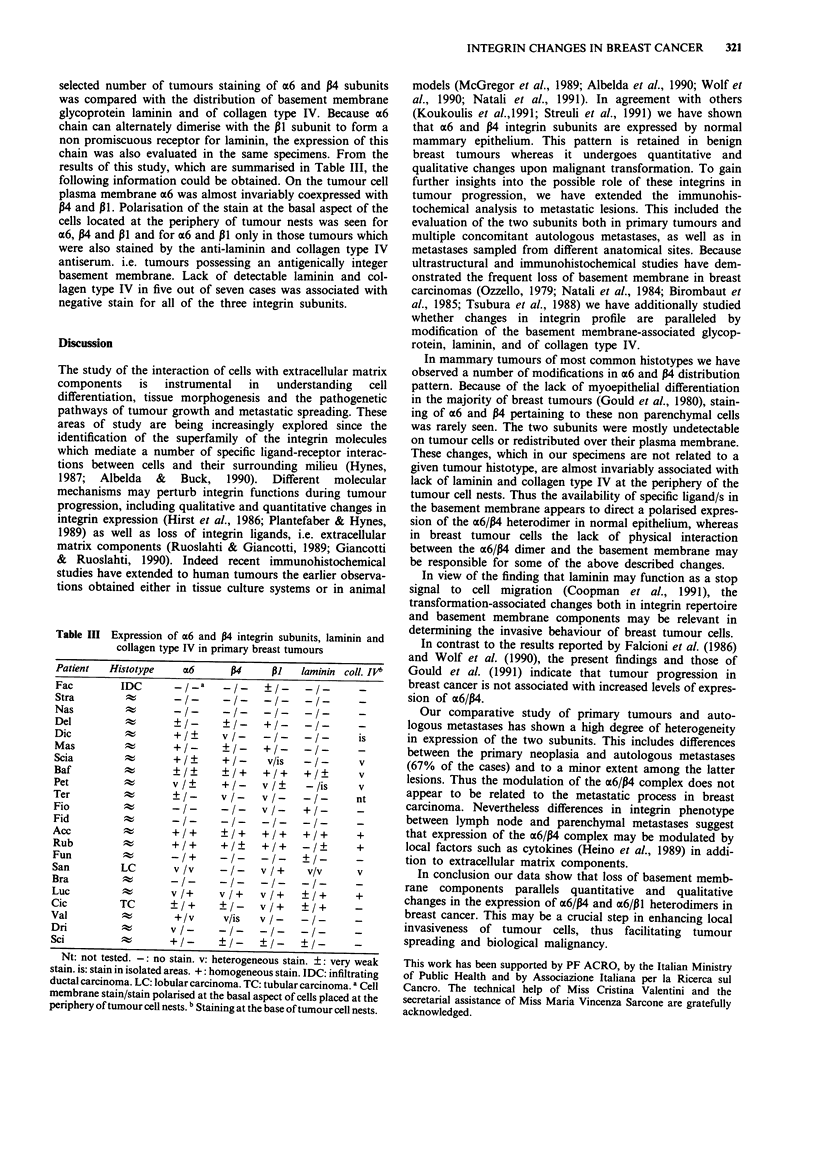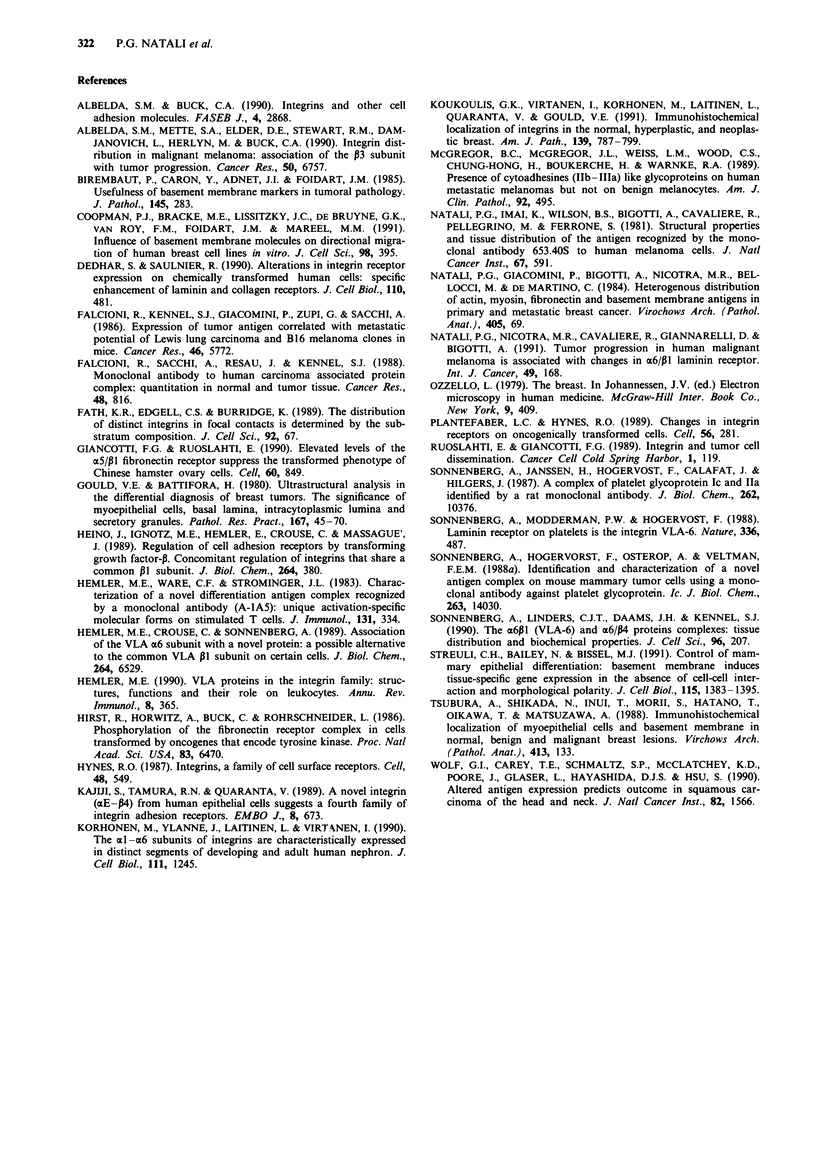# Changes in expression of alpha 6/beta 4 integrin heterodimer in primary and metastatic breast cancer.

**DOI:** 10.1038/bjc.1992.263

**Published:** 1992-08

**Authors:** P. G. Natali, M. R. Nicotra, C. Botti, M. Mottolese, A. Bigotti, O. Segatto

**Affiliations:** Regina Elena Cancer Institute, Rome, Italy.

## Abstract

**Images:**


Br. J. Cancer (1992), 66, 318-322 © Macmillan Press Ltd., 1992

### Changes in expression of α6/β4 integrin heterodimer in primary and metastatic breast cancer

P.G. Natali[1], M.R. Nicotra[2], C. Botti[1], M. Mottolese[1], A. Bigotti[1] & O. Segatto[1]

[1]*Regina Elena Cancer Institute, Rome;* [2]*Ist. Biomedical Technologies, CNR, Rome, Italy.*

**Summary** The α6/β4 integrin complex has been shown to be expressed in murine tissues at the basolateral aspect of most epithelial cells including the mammary epithelium, thus suggesting that this heterodimer may interact with components of the basement membrane. Because transformation of mammary epithelium frequently results in disappearance of basement membranes and loss of cell polarisation we have analysed in the present study whether expression of the α6/β4 complex is altered in human breast tumours. The results of the present study confirm that in human mammary gland α6 and β4 subunits colocalise at the basolateral aspect of the epithelium. While in benign breast lesions this distribution pattern remains mostly unchanged, in primary carcinomas the expression of both chains is either redistributed over the cell surface or significantly reduced. This altered pattern of expression is paralleled by the lack of detection of basement membrane laminin and collagen type IV. In metastatic lesions the expression of the heterodimer is maintained in most of the lymphonodal foci, but less frequently detected in metastasis localised in the pleural cavity and in parenchymal tissues. These findings indicate that in breast epithelium expression of the α6/β4 heterodimer is modulated by the presence of basement membrane and is possibly influenced by microenvironmental factors as suggested by the different pattern of α6/β4 expression in nodal and extranodal metastatic foci.

Integrins represent an expanding family of heterodimeric receptors (Hynes, 1987) involved in cell-to-cell and cell matrix interactions (Albelda & Buck, 1990). Accumulating experimental evidence points to a major functional role of integrins in the regulation of cell polarity (Fath *et al.*, 1989) and migration (Hemler, 1990) as well as in morphogenesis (Korhonen *et al.*, 1990). It has also been proposed that derangement of integrin expression may be responsible for a number of aberrant cell behaviours during tumour onset, progression and metastatic spreading (Plantefaber & Hynes, 1989; Ruoslahti & Giancotti, 1989; Dedhar & Saulnier, 1990; Giancotti & Ruoslahti, 1990).

In this context the VLA6 (Sonnenberg *et al.*, 1987) integrin which is formed by the non covalent association of α6 and β1 chains is of particular interest since it represents a non promiscuous receptor for the basement membrane glycoprotein laminin (Sonnenberg *et al.*, 1988). However, the α6 chain can alternatively associate with a different β chain to form the α6/β4 heterodimer (Sonnenberg *et al.*, 1988*a*; Hemler *et al.*, 1989; Kajiji *et al.*, 1989) whose receptorial activity is not yet fully characterised.

Detailed immunohistochemical studies of murine tissues (Sonnenberg *et al.*, 1990) have revealed that α6, β4 and β1 codistribute in most epithelia including the mammary epithelium at the basolateral aspect, thus suggesting that α6/β4 dimers physically interact with some basement membrane component/s which may in turn modulate this expression and cellular compartimentalisation (Fath *et al.*, 1989). The observation that transformation of mammary epithelium is frequently associated with lack of basement membranes (Ozzello, 1979; Natali *et al.*, 1984; Birembaut *et al.*, 1985; Tsubura *et al.*, 1988) provides the opportunity to test this hypothesis through the comparative analysis of α6/β4 expression in normal and transformed primary and metastatic human mammary epithelium.

We report here that in human breast tumours the lack of laminin and collagen type IV i.e. basement membranes is associated with a significantly reduced expression of α6/β4 as well as loss of its polarised pattern of expression.

Correspondence: P. Giorgio Natali, Regina Elena Cancer Institute, Immunology Laboratory, Via delle Messi d'Oro 156-158, 00158 Rome, Italy.
Received 25 November 1991; and in revised form 27 April 1992.

### Materials and methods

### *Tissues*

Surgical biopsies of normal, benign and malignant tumour tissues were collected following ablative surgery from patients free of chemo and radiotherapy. Tissues were snap frozen in liquid nitrogen. From each specimens consecutive 4 μ cryostat sections were obtained which were fixed in cold absolute acetone for 10 min. Fixed sections were either immediately used in immunohistochemical assay or kept frozen at − 30°C with no loss of serological activity. Fixed sections stained with 1% toluidine blue were used to evaluate the histological features of the tissues.

### *Monoclonal and polyclonal antisera*

The murine monoclonal antibody (MoAb) A-1A5 to the β1 subunit (Hemler *et al.*, 1983) was kindly provided by Dr M.E. Hemler (Dana Farber Cancer Inst., Boston Ma., USA). The rat MoAb 135-13C to the α6 (Falcioni *et al.*, 1986) and MoAb 439-9B (Falcioni *et al.*, 1988) to the β4 integrin subunits were kindly supplied by Dr A. Sacchi (Laboratory of Molecular Oncogenesis, Regina Elena Cancer Inst., Rome, Italy). Commercially available murine MoAb to α6 (HP2/1) and β4 (3EI) were from Immunotech (Marseille, France) and Telios Pharmaceutical Inc. (San Diego, Ca. USA) respectively. Rabbit anti-laminin antiserum was purchased from Chemicon Int. (El Segundo, Ca., USA). Murine monoclonal antibodies to collagen type IV were purchased from Sigma Chemical (St Louis. Mo. USA).

### *Immunohistochemical assay*

Indirect immunoperoxidase (IIP) staining was performed by employing on consecutive sections of the same specimen primary MoAbs (25 to 50 $\mu g\,ml^{-1}$) and a commercially available avidin-biotin staining kits (Vector Lab., Burlingame, Ca., USA). Because the affinity of MoAbs was unknown the incubation with tissue sections was prolonged for 18 h. Negative controls consisted of tissue sections incubated with irrelevant MoAb. The positive stain of the vascular walls observable with antibodies provided a positive control in each specimen studied. The immunoenzymatic reaction employed 3-amino-9-ethylcarbazole as a chromogenic substrate and Mayer's hematoxylin as nuclear counterstain followed by mounting in buffered glycerol. Indirect immunofluorescence was done as described (Natali *et al.*, 1981) using MoAb at the concentration of 25 $\mu g\,ml^{-1}$.

### Results

### *Expression of α6 and β4 subunits in normal mammary epithelium and benign breast lesions*

Immunohistochemical analysis of normal breast tissue revealed a consistently strong stain for α6 and β4 which outlined the outer aspect of acini and ducts independently from the discontinuous (acini) and continuous (ducts) distribution of myoepithelial cells. A heterogenous stain of the lateral aspect of luminal cells was seen with MoAb 135.13C to α6 and was, even more pronounced with antibodies to β4 chain. Staining of α6 and β4 at the basal aspect of luminal cells was rarely seen. By indirect immunofluorescence which in our hands allowed a higher resolution, an ordered punctuate stain could be observed for α6 and β4 in section planes running tangential to the basal portion of the ductal and acinar epithelium (Figure 1a inset). The extent to which myoepithelial and luminal cells contributed to this pattern could not be firmly established. The staining patterns described above were maintained in three types of benign breast tumours tested (Table I). Only in two cases of gynecomastia was the plasma-membrane stain for α6 not associated with detectable levels of β4.

### *Changes in distribution of α6 and β4 subunits in primary and metastatic breast tumours*

Evaluation of primary breast tumours of the most common histotypes (Table I) indicates that the expression of α6 and β4 subunits undergoes a number of changes. As a general rule, β4 was never expressed in absence of α6. Three major staining patterns were observed. Staining for α6 and β4 in a significant number of tumours was undetectable at the level of the cell membrane (Figure 1b). This was more frequently seen in lobular and infiltrating ductal carcinomas while it was less common in tubular tumours. Moreover polarised stain for both subunits at the periphery of tumour cell nests (Figure 1c) was rare in most tumour histotypes. The punctuate stain at the base of tumour cell nests was never observed.

The results of the comparative immunohistochemical evaluation of primary tumours and autologous metastasis as well as of metastasis from various anatomical sites are summarised in Table II. Also in this instance three major staining patterns could be observed since staining for β4 was never observed in the absence of detectable α6 chain. Among metastatic lesions, especially those located in lymph nodes (40%) displayed stain for both subunits on tumour cell membrane (Figure 1d). In only four out of 26 metastases was a polarisation of the stain for both chains seen at the periphery of tumour cell nests. While the distribution of both subunits in primary lesions was often (67%) different from that observed in metastatic foci, the distribution of both chains was rather consistent among multiple concomitant autologous metastases. In one case (patient Br) whilst the primary tumour lacked α6 and β4 stain, both chains were expressed in the lymphonodal autologous lesions. As opposed to nodal lesions, parenchymal and particularly pleural metastasis (ten

**Figure 1** Immunohistochemical distribution of α6/β4 integrin subunits in normal and transformed mammary epithelium. MoAb 135-13C to the α6 subunit decorates the basolateral aspect of normal ductal cells **a**. The stain appears more intense at the basal region where by indirect immunofluorescence a fine row of punctate reaction may be seen (inset). **b**, Shows both normal (large arrows) and transformed (small arrows) epithelium (IDC). The β4 subunit is present in the myoepithelial layer (large arrow) of normal ducts and is variably expressed at the periphery of tumour cell nests (small arrows). α6 expression is maintained with a normal pattern of distribution in a case of IDC **c**, Cells of a lymphonodal metastasis **d**, are heterogeneously reactive with MoAb 135-13C to the α6 chain. Indirect avidin-biotin immunoperoxidase. Counterstain Mayer's haematoxylin. (**a**–**c**, bar = 30 μ; **d**, bar = 20 μ).

**Table I** Pattern of expression of α6 and β4 integrin subunits in benign and malignant mammary lesions

| | | *Expression patterns* α6( + ) β4( + ) | | α6( + ) β4( − ) | | α6( − ) β4( − ) | |
|---|---|---|---|---|---|---|---|
| *Malignant* | | cell | basal | cell | basal | cell | basal |
| IDC (27)[a] | | 9[b] | 3 | 6 | 4 | 12 | 20 |
| LC (14) | | 3 | 4 | 2 | 3 | 9 | 7 |
| TC (8) | | 5 | 2 | 2 | 2 | 1 | 4 |
| *Benign* | | | | | | | |
| Fibrocystic | (6) | 6 | 6 | | | | |
| Fibroadenoma | (7) | 7 | 7 | | | | |
| Gynecomastia | (5) | 3 | 5 | 2 | | | |

[a]Number of cases tested. [b]Number of cases with a given staining pattern. IDC: infiltrating ductal carcinoma. LC: lobular carcinoma. TC: tubular carcinoma. Cell: expression on the cell surface. Basal: polarised expression at the basal cell aspect of cell placed at the periphery of tumour cell nests.

**Table II** Pattern of expression of α6/β4 intregrin in metastatic breast cancer

| *Case* | *Lesion* | *Histotype* | *Expression patterns* α6( + )β4( + ) | α6( + )β4( − ) | α6( − )β4( − ) |
|---|---|---|---|---|---|
| DC | P | IDC | + / ±[a] | | |
| | M1 (Ly) | | ± / ± | | |
| | M2 ≈ | | | | − / − |
| | M3 ≈ | | v/ − | | |
| | M4 ≈ | | | | − / − |
| MA | P | IDC | v/v | | |
| | M1 (Ly) | | + / − | | |
| | M2 ≈ | | + / − | | |
| | M3 ≈ | | + / − | | |
| DS | P | TC | + / ± | | |
| | M1 (Ly) | | | + / − | |
| | M2 ≈ | | | + / − | |
| | M3 ≈ | | | + / − | |
| BR | P | LC | | | − / − |
| | M1 (Ly) | | + / − | | |
| | M2 ≈ | | ± / − | | |
| BO | P | LC | | | − / − |
| | M1 (Ly) | | | ± / − | |
| | M2 ≈ | | | | − / − |
| SA | P | LC | | ± / − | |
| | M1 (Ly) | | | ± / − | |
| ZA | P | LC | ± / ± | | |
| | M1 (Ly) | | | ± / − | |
| PE | P | IDC | | ± / − | |
| | M1 (Ly) | | | + / − | |
| TE | P | IDC | | + / − | |
| | M1 (Ly) | | | + / − | |
| | M2 ≈ | | | + / − | |
| | M3 ≈ | | | + / − | |
| CA | M (Pu) | IDC | | | − / − |
| DO | M (Sc) | | | | − / − |
| DI | M (Ce) | | + / + | | |
| FA | M (Pu) | | | | − / − |
| ST | M (Sc) | | + / − | | |
| DM | M (Pu) | | | | − / − |

P: primary tumour. M: individual concomitant metastasis. Ly: lymphonodal. Pu: pulmonary. Sc: subcutaneous. Ce: cerebral. IDC: infiltrating ductal carcinoma. TC: tubular carcinoma. LC: lobular carcinoma. + : homogeneous stain. ± : very weak stain. v: stain of heterogeneous intensity. − : no stain. [a] Cell stain/stain polarised at the basal aspect of cells placed at the periphery of tumour cell nests.

cases not shown) were found to be negative for α6 and β4 stain over a wide range of MoAb concentrations. Stain for β1 subunit performed in four of these lesions was however consistently positive. All the described staining patterns remained unchanged when using additional MoAb HP2/1 and 3E1 to the α6 and β4 chains respectively.

*Relationship between integrin phenotype and basement membrane antigens in primary breast tumours*

In order to assess whether the changes in expression and cellular compartmentalisation of the α6 and β4 subunits observed in primary mammary tumours might be associated with an altered distribution of basement membrane, in a selected number of tumours staining of α6 and β4 subunits was compared with the distribution of basement membrane glycoprotein laminin and of collagen type IV. Because α6 chain can alternately dimerise with the β1 subunit to form a non promiscuous receptor for laminin, the expression of this chain was also evaluated in the same specimens. From the results of this study, which are summarised in Table III, the following information could be obtained. On the tumour cell plasma membrane α6 was almost invariably coexpressed with β4 and β1. Polarisation of the stain at the basal aspect of the cells located at the periphery of tumour nests was seen for α6, β4 and β1 and for α6 and β1 only in those tumours which were also stained by the anti-laminin and collagen type IV antiserum. i.e. tumours possessing an antigenically integer basement membrane. Lack of detectable laminin and collagen type IV in five out of seven cases was associated with negative stain for all of the three integrin subunits.

### Discussion

The study of the interaction of cells with extracellular matrix components is instrumental in understanding cell differentiation, tissue morphogenesis and the pathogenetic pathways of tumour growth and metastatic spreading. These areas of study are being increasingly explored since the identification of the superfamily of the integrin molecules which mediate a number of specific ligand-receptor interactions between cells and their surrounding milieu (Hynes, 1987; Albelda & Buck, 1990). Different molecular mechanisms may perturb integrin functions during tumour progression, including qualitative and quantitative changes in integrin expression (Hirst *et al.*, 1986; Plantefaber & Hynes, 1989) as well as loss of integrin ligands, i.e. extracellular matrix components (Ruoslahti & Giancotti, 1989; Giancotti & Ruoslahti, 1990). Indeed recent immunohistochemical studies have extended to human tumours the earlier observations obtained either in tissue culture systems or in animal models (McGregor *et al.*, 1989; Albelda *et al.*, 1990; Wolf *et al.*, 1990; Natali *et al.*, 1991). In agreement with others (Koukoulis *et al.*,1991; Streuli *et al.*, 1991) we have shown that α6 and β4 integrin subunits are expressed by normal mammary epithelium. This pattern is retained in benign breast tumours whereas it undergoes quantitative and qualitative changes upon malignant transformation. To gain further insights into the possible role of these integrins in tumour progression, we have extended the immunohistochemical analysis to metastatic lesions. This included the evaluation of the two subunits both in primary tumours and multiple concomitant autologous metastases, as well as in metastases sampled from different anatomical sites. Because ultrastructural and immunohistochemical studies have demonstrated the frequent loss of basement membrane in breast carcinomas (Ozzello, 1979; Natali *et al.*, 1984; Birombaut *et al.*, 1985; Tsubura *et al.*, 1988) we have additionally studied whether changes in integrin profile are paralleled by modification of the basement membrane-associated glycoprotein, laminin, and of collagen type IV.

**Table III** Expression of α6 and β4 integrin subunits, laminin and collagen type IV in primary breast tumours

| *Patient* | *Histotype* | *α6* | *β4* | *β1* | *laminin* | *coll. IV*[b] |
|---|---|---|---|---|---|---|
| Fac | IDC | −/−[a] | −/− | ±/− | −/− | − |
| Stra | ≈ | −/− | −/− | −/− | −/− | − |
| Nas | ≈ | −/− | −/− | −/− | −/− | − |
| Del | ≈ | ±/− | ±/− | +/− | −/− | − |
| Dic | ≈ | +/± | v/− | −/− | −/− | is |
| Mas | ≈ | +/− | ±/− | +/− | −/− | − |
| Scia | ≈ | +/± | +/− | v/is | −/− | v |
| Baf | ≈ | ±/± | ±/+ | +/+ | +/± | v |
| Pet | ≈ | v/± | +/− | v/± | −/is | v |
| Ter | ≈ | ±/− | v/− | v/− | −/− | nt |
| Fio | ≈ | −/− | −/− | v/− | +/− | − |
| Fid | ≈ | −/− | −/− | −/− | −/− | − |
| Acc | ≈ | +/+ | ±/+ | +/+ | +/+ | + |
| Rub | ≈ | +/+ | +/± | +/+ | −/± | + |
| Fun | ≈ | −/+ | −/− | −/− | ±/− | − |
| San | LC | v/v | −/− | v/+ | v/v | v |
| Bra | ≈ | −/− | −/− | −/− | −/− | − |
| Luc | ≈ | v/+ | v/+ | v/+ | ±/+ | + |
| Cic | TC | ±/+ | ±/− | v/+ | ±/+ | − |
| Val | ≈ | +/v | v/is | v/− | −/− | − |
| Dri | ≈ | v/− | −/− | −/− | −/− | − |
| Sci | ≈ | +/− | ±/− | ±/− | ±/− | − |

Nt: not tested. −: no stain. v: heterogeneous stain. ±: very weak stain. is: stain in isolated areas. +: homogeneous stain. IDC: infiltrating ductal carcinoma. LC: lobular carcinoma. TC: tubular carcinoma. [a] Cell membrane stain/stain polarised at the basal aspect of cells placed at the periphery of tumour cell nests. [b] Staining at the base of tumour cell nests.

In mammary tumours of most common histotypes we have observed a number of modifications in α6 and β4 distribution pattern. Because of the lack of myoepithelial differentiation in the majority of breast tumours (Gould *et al.*, 1980), staining of α6 and β4 pertaining to these non parenchymal cells was rarely seen. The two subunits were mostly undetectable on tumour cells or redistributed over their plasma membrane. These changes, which in our specimens are not related to a given tumour histotype, are almost invariably associated with lack of laminin and collagen type IV at the periphery of the tumour cell nests. Thus the availability of specific ligand/s in the basement membrane appears to direct a polarised expression of the α6/β4 heterodimer in normal epithelium, whereas in breast tumour cells the lack of physical interaction between the α6/β4 dimer and the basement membrane may be responsible for some of the above described changes.

In view of the finding that laminin may function as a stop signal to cell migration (Coopman *et al.*, 1991), the transformation-associated changes both in integrin repertoire and basement membrane components may be relevant in determining the invasive behaviour of breast tumour cells.

In contrast to the results reported by Falcioni *et al.* (1986) and Wolf *et al.* (1990), the present findings and those of Gould *et al.* (1991) indicate that tumour progression in breast cancer is not associated with increased levels of expression of α6/β4.

Our comparative study of primary tumours and autologous metastases has shown a high degree of heterogeneity in expression of the two subunits. This includes differences between the primary neoplasia and autologous metastases (67% of the cases) and to a minor extent among the latter lesions. Thus the modulation of the α6/β4 complex does not appear to be related to the metastatic process in breast carcinoma. Nevertheless differences in integrin phenotype between lymph node and parenchymal metastases suggest that expression of the α6/β4 complex may be modulated by local factors such as cytokines (Heino *et al.*, 1989) in addition to extracellular matrix components.

In conclusion our data show that loss of basement membrane components parallels quantitative and qualitative changes in the expression of α6/β4 and α6/β1 heterodimers in breast cancer. This may be a crucial step in enhancing local invasiveness of tumour cells, thus facilitating tumour spreading and biological malignancy.

This work has been supported by PF ACRO, by the Italian Ministry of Public Health and by Associazione Italiana per la Ricerca sul Cancro. The technical help of Miss Cristina Valentini and the secretarial assistance of Miss Maria Vincenza Sarcone are gratefully acknowledged.

### References

ALBELDA, S.M. & BUCK, C.A. (1990). Integrins and other cell adhesion molecules. *FASEB J.*, **4**, 2868.

ALBELDA, S.M., METTE, S.A., ELDER, D.E., STEWART, R.M., DAMJANOVICH, L., HERLYN, M. & BUCK, C.A. (1990). Integrin distribution in malignant melanoma: association of the $\beta3$ subunit with tumor progression. *Cancer Res.*, **50**, 6757.

BIREMBAUT, P., CARON, Y., ADNET, J.I. & FOIDART, J.M. (1985). Usefulness of basement membrane markers in tumoral pathology. *J. Pathol.*, **145**, 283.

COOPMAN, P.J., BRACKE, M.E., LISSITZKY, J.C., DE BRUYNE, G.K., VAN ROY, F.M., FOIDART, J.M. & MAREEL, M.M. (1991). Influence of basement membrane molecules on directional migration of human breast cell lines *in vitro*. *J. Cell Sci.*, **98**, 395.

DEDHAR, S. & SAULNIER, R. (1990). Alterations in integrin receptor expression on chemically transformed human cells: specific enhancement of laminin and collagen receptors. *J. Cell Biol.*, **110**, 481.

FALCIONI, R., KENNEL, S.J., GIACOMINI, P., ZUPI, G. & SACCHI, A. (1986). Expression of tumor antigen correlated with metastatic potential of Lewis lung carcinoma and B16 melanoma clones in mice. *Cancer Res.*, **46**, 5772.

FALCIONI, R., SACCHI, A., RESAU, J. & KENNEL, S.J. (1988). Monoclonal antibody to human carcinoma associated protein complex: quantitation in normal and tumor tissue. *Cancer Res.*, **48**, 816.

FATH, K.R., EDGELL, C.S. & BURRIDGE, K. (1989). The distribution of distinct integrins in focal contacts is determined by the substratum composition. *J. Cell Sci.*, **92**, 67.

GIANCOTTI, F.G. & RUOSLAHTI, E. (1990). Elevated levels of the $\alpha5/\beta1$ fibronectin receptor suppress the transformed phenotype of Chinese hamster ovary cells. *Cell*, **60**, 849.

GOULD, V.E. & BATTIFORA, H. (1980). Ultrastructural analysis in the differential diagnosis of breast tumors. The significance of myoepithelial cells, basal lamina, intracytoplasmic lumina and secretory granules. *Pathol. Res. Pract.*, **167**, 45–70.

HEINO, J., IGNOTZ, M.E., HEMLER, E., CROUSE, C. & MASSAGUE', J. (1989). Regulation of cell adhesion receptors by transforming growth factor-$\beta$. Concomitant regulation of integrins that share a common $\beta1$ subunit. *J. Biol. Chem.*, **264**, 380.

HEMLER, M.E., WARE, C.F. & STROMINGER, J.L. (1983). Characterization of a novel differentiation antigen complex recognized by a monoclonal antibody (A-1A5): unique activation-specific molecular forms on stimulated T cells. *J. Immunol.*, **131**, 334.

HEMLER, M.E., CROUSE, C. & SONNENBERG, A. (1989). Association of the VLA $\alpha6$ subunit with a novel protein: a possible alternative to the common VLA $\beta1$ subunit on certain cells. *J. Biol. Chem.*, **264**, 6529.

HEMLER, M.E. (1990). VLA proteins in the integrin family: structures, functions and their role on leukocytes. *Annu. Rev. Immunol.*, **8**, 365.

HIRST, R., HORWITZ, A., BUCK, C. & ROHRSCHNEIDER, L. (1986). Phosphorylation of the fibronectin receptor complex in cells transformed by oncogenes that encode tyrosine kinase. *Proc. Natl Acad. Sci. USA*, **83**, 6470.

HYNES, R.O. (1987). Integrins, a family of cell surface receptors. *Cell*, **48**, 549.

KAJIJI, S., TAMURA, R.N. & QUARANTA, V. (1989). A novel integrin ($\alpha$E–$\beta4$) from human epithelial cells suggests a fourth family of integrin adhesion receptors. *EMBO J.*, **8**, 673.

KORHONEN, M., YLANNE, J., LAITINEN, L. & VIRTANEN, I. (1990). The $\alpha1$–$\alpha6$ subunits of integrins are characteristically expressed in distinct segments of developing and adult human nephron. *J. Cell Biol.*, **111**, 1245.

KOUKOULIS, G.K., VIRTANEN, I., KORHONEN, M., LAITINEN, L., QUARANTA, V. & GOULD, V.E. (1991). Immunohistochemical localization of integrins in the normal, hyperplastic, and neoplastic breast. *Am. J. Path.*, **139**, 787–799.

MCGREGOR, B.C., MCGREGOR, J.L., WEISS, L.M., WOOD, C.S., CHUNG-HONG, H., BOUKERCHE, H. & WARNKE, R.A. (1989). Presence of cytoadhesines (IIb–IIIa) like glycoproteins on human metastatic melanomas but not on benign melanocytes. *Am. J. Clin. Pathol.*, **92**, 495.

NATALI, P.G., IMAI, K., WILSON, B.S., BIGOTTI, A., CAVALIERE, R., PELLEGRINO, M. & FERRONE, S. (1981). Structural properties and tissue distribution of the antigen recognized by the monoclonal antibody 653.40S to human melanoma cells. *J. Natl Cancer Inst.*, **67**, 591.

NATALI, P.G., GIACOMINI, P., BIGOTTI, A., NICOTRA, M.R., BELLOCCI, M. & DE MARTINO, C. (1984). Heterogenous distribution of actin, myosin, fibronectin and basement membrane antigens in primary and metastatic breast cancer. *Virochows Arch. (Pathol. Anat.)*, **405**, 69.

NATALI, P.G., NICOTRA, M.R., CAVALIERE, R., GIANNARELLI, D. & BIGOTTI, A. (1991). Tumor progression in human malignant melanoma is associated with changes in $\alpha6/\beta1$ laminin receptor. *Int. J. Cancer*, **49**, 168.

OZZELLO, L. (1979). The breast. In Johannessen, J.V. (ed.) Electron microscopy in human medicine. *McGraw-Hill Inter. Book Co., New York*, **9**, 409.

PLANTEFABER, L.C. & HYNES, R.O. (1989). Changes in integrin receptors on oncogenically transformed cells. *Cell*, **56**, 281.

RUOSLAHTI, E. & GIANCOTTI, F.G. (1989). Integrin and tumor cell dissemination. *Cancer Cell Cold Spring Harbor*, **1**, 119.

SONNENBERG, A., JANSSEN, H., HOGERVOST, F., CALAFAT, J. & HILGERS, J. (1987). A complex of platelet glycoprotein Ic and IIa identified by a rat monoclonal antibody. *J. Biol. Chem.*, **262**, 10376.

SONNENBERG, A., MODDERMAN, P.W. & HOGERVOST, F. (1988). Laminin receptor on platelets is the integrin VLA-6. *Nature*, **336**, 487.

SONNENBERG, A., HOGERVORST, F., OSTEROP, A. & VELTMAN, F.E.M. (1988*a*). Identification and characterization of a novel antigen complex on mouse mammary tumor cells using a monoclonal antibody against platelet glycoprotein. *Ic. J. Biol. Chem.*, **263**, 14030.

SONNENBERG, A., LINDERS, C.J.T., DAAMS, J.H. & KENNEL, S.J. (1990). The $\alpha6\beta1$ (VLA-6) and $\alpha6/\beta4$ proteins complexes: tissue distribution and biochemical properties. *J. Cell Sci.*, **96**, 207.

STREULI, C.H., BAILEY, N. & BISSEL, M.J. (1991). Control of mammary epithelial differentiation: basement membrane induces tissue-specific gene expression in the absence of cell-cell interaction and morphological polarity. *J. Cell Biol.*, **115**, 1383–1395.

TSUBURA, A., SHIKADA, N., INUI, T., MORII, S., HATANO, T., OIKAWA, T. & MATSUZAWA, A. (1988). Immunohistochemical localization of myoepithelial cells and basement membrane in normal, benign and malignant breast lesions. *Virchows Arch. (Pathol. Anat.)*, **413**, 133.

WOLF, G.I., CAREY, T.E., SCHMALTZ, S.P., MCCLATCHEY, K.D., POORE, J., GLASER, L., HAYASHIDA, D.J.S. & HSU, S. (1990). Altered antigen expression predicts outcome in squamous carcinoma of the head and neck. *J. Natl Cancer Inst.*, **82**, 1566.